# Prolonged Cryopreservation Negatively Affects Embryo Transfer Outcomes Following the Elective Freeze-All Strategy: A Multicenter Retrospective Study

**DOI:** 10.3389/fendo.2021.709648

**Published:** 2021-09-22

**Authors:** Xudong Zhang, Shanshan Wu, Guimin Hao, Xueqing Wu, Haiqin Ren, Yinfeng Zhang, Aimin Yang, Xingyu Bi, Lina Bai, Yunshan Zhang, Jichun Tan

**Affiliations:** ^1^Center of Reproductive Medicine, Department of Obstetrics and Gynecology, Shengjing Hospital of China Medical University, Shenyang, China; ^2^Key Laboratory of Reproductive Dysfunction Disease and Fertility Remodeling of Liaoning Province, Shenyang, China; ^3^Department of Reproductive Medicine, The Second Hospital of Hebei Medical University, Shijiazhuang, China; ^4^Center of Reproductive Medicine, Children’s Hospital of Shanxi and Women Health Center of Shanxi, Taiyuan, China; ^5^Center of Reproductive Medicine, Jinghua Hospital, Shenyang, China; ^6^Center for Reproductive Medicine, Tianjin Central Hospital of Obstetrics and Gynecology, Tianjin, China

**Keywords:** assisted reproductive technology, frozen-thawed embryo transfer, freeze-all, cryopreservation, reproductive outcomes

## Abstract

**Background:**

With the development of embryo freezing and warming technology, frozen-thawed embryo transfer (FET) has been widely utilized. However, studies investigating the association between cryopreservation duration and FET outcomes are limited and controversial, and previous studies did not conduct stratification analyses based on demographic or clinical characteristics.

**Methods:**

This multicenter retrospective study included 17,826 women who underwent their first FET following the freeze-all strategy during the period from January 2014 to December 2018. Duration of cryopreservation was categorized into five groups: 3–8 weeks, 8–12 weeks, 12–26 weeks, 26–52 weeks, and >52 weeks. Modified Poisson regression and multivariate logistic regression were used to assess the association between cryostorage time of vitrified embryos and transfer outcomes. Moreover, further stratification analyses were performed according to variables with *p <*0.05 in multivariate models.

**Results:**

In this large multicenter study, we observed that storage duration was inversely associated with the possibility of pregnancy and live birth (*p <*0.001), but not with the risk of ectopic pregnancy and miscarriage. Stratification analyses based on maternal age, the number of oocytes retrieved, and condition of embryo transferred indicated that the inverse correlation was significant in the subpopulation with characteristics: (1) less than 40 years old, (2) more than 3 oocytes retrieved, and (3) only high-quality blastocysts transferred.

**Conclusion:**

The results of this large, multicenter, retrospective study suggested that prolonged cryopreservation was inversely associated with the probability of pregnancy and live birth. Therefore, for patients who adopt a freeze-all strategy, early FET might achieve a better outcome.

## Introduction

In recent years, innovations in freezing technology from ‘slow freezing’ to ‘vitrification freezing’ have attracted widespread attention. Compared to slow freezing, vitrification may significantly reduce the formation of ice crystals inside and outside cells during embryo freezing, thereby minimizing the extent of embryo damage and risk of developmental arrest (1). *In vitro* experiments have shown that vitrification can improve embryo survival after recovery by reducing DNA apoptosis and maintaining DNA integrity (2, 3). In addition, a systematic review suggested that vitrification was significantly better than slow freezing in terms of embryo survival rates after resuscitation and pregnancy outcomes (4).

With the development of embryo freezing and warming technology, frozen-thawed embryo transfer (FET) has been widely utilized. According to data from the Chinese Society of Reproductive Medicine (CSRM), in 2016, 133 reproductive centers in China implemented about 151,889 FET cycles, accounting for more than 40% of the total number of assisted reproductive technology (ART) cycles (5). Studies have shown that FET can improve cumulative pregnancy and live birth rates within a single ovarian stimulation cycle, avoid the repeated use of ovulation-inducing drugs and the risks associated with oocyte retrieval procedures, and save costs for patients (1, 6).

Ovulation stimulation protocols may have adverse effects on endometrial receptivity, and hence hinder the implantation of embryos (7), thus elective freeze-all strategy was introduced to overcome this problem. Elective freeze-all strategy refers to freezing all viable embryos in an ovarian stimulation cycle for subsequent FET (8). Previous studies demonstrated that it can improve embryo-endometrial synchronization, allowing embryo transfer to be implemented within an implantation window closer to the physiological state, and reduce the incidence of ovulation hyperstimulation syndrome (OHSS) (9).

Studies have determined that FET followed by the freeze-all policy did not improve live birth rates for women with normal ovarian response, but significantly improved live birth rates and reduced pregnancy loss rates in individuals with the polycystic ovarian syndrome (PCOS)/ovarian hyperresponsiveness (10, 11). Therefore, the freeze-all strategy should be implemented individually. Its main indications include patients at high risk of OHSS, PCOS/ovarian hyperresponsiveness, the requirement for preimplantation genetic diagnosis/*s*creening (PGD/PGS), late-follicular phase elevated serum progesterone levels, endometriosis/adenomyosis, and recurrent implantation failure due to defective endometrial receptivity (12). Further studies are necessary to determine the long-term associated risks of the freeze-all strategy and the specific subpopulation that are more likely to benefit from it.

Although live birth has been reported after the transfer of frozen-thawed embryos that have been cryopreserved for up to 20 years (13), the effects of long-term, ultra-low temperature preservation on embryos’ implantation potential and pregnancy outcomes are inconclusive. Previous studies indicated that prolonged frozen storage time did not affect pregnancy outcomes (14–18). On the contrary, a recent large-population study suggested that cryopreservation duration may be negatively correlated with pregnancy and live birth rates (19). Moreover, previous studies did not conduct stratification analyses based on demographic or clinical characteristics to further assess the impact of frozen storage time on FET outcomes. Given these, we conducted a large, multicenter, retrospective cohort study to investigate the effect of cryopreservation duration on reproductive outcomes among 17,826 women who underwent their first FET cycle following the freeze-all strategy.

## Materials and Methods

### Study Population

This multicenter retrospective study was conducted in four reproductive centers in northern China and included 17,826 women who underwent their first FET cycle following the freeze-all strategy from January 2014 to December 2018. Cycles that used PGD/PGS, donor oocytes, donor sperm, and transferred mixed-stage embryos, or had no available embryo for transfer were excluded. The primary outcome was live birth, and the secondary outcomes included biochemical pregnancy, clinical pregnancy, ectopic pregnancy, and miscarriage. This study was approved by the Ethics Committee of Shengjing Hospital of China Medical University (2020PS011F). All data used in this study were anonymous and did not have any identifiers. Written informed consent for this study was not required in accordance with local legislation and national guidelines.

### ART Procedures

ART procedures have been described in detail in previous studies (20–22) and include four stages: (1) ovulation induction; (2) oocyte retrieval; (3) embryo freezing, thawing, and transfer; and (4) pregnancy test. Human chorionic gonadotropin (hCG) was administered to induce ovulation when the diameters of three or more leading follicles reached 18 mm. Oocyte retrieval was performed 34–36 h after hCG injection. Fresh semen was obtained on the same day by masturbation after 2–7 days of abstinence. Semen samples were handled and analyzed according to the World Health Organization (2010) recommendations and prepared for fertilization using a density gradient centrifugation step. Retrieved oocyte–cumulus complexes were fertilized using *in vitro* fertilization (IVF) or intracytoplasmic sperm injection (ICSI) upon clinical indication.

Normal fertilization was evidenced by the presence of two pronuclei. The number of oocytes fertilized normally and the quality of embryos formed were evaluated by embryologists 16–18 h after injection. On day 3, the Peter scoring system was used to assess the quality of embryos based on the size, shape, and fragmentation of blastomeres (23). Embryos with 6‒10 cells, even size, regular shape, and <20% fragmentation were considered as good-quality embryos (24). At the blastocyst stage, embryos were evaluated using the Gardner system (25): (1) blastocysts were rated as grades 1‒6 according to the degree of blastocyst expansion and hatching, and (2) for blastocysts graded as 3–6, further A–C scores were assigned based on the number and cohesiveness of the inner cell mass and trophectoderm. The high-quality blastocyst was defined as that of grade ≥3BB on day 5 or ≥4BB on day 6 (26).

Cleavage-stage embryos were vitrified on day 3 and blastocysts were vitrified on day 5 or 6 according to embryo development. Vitrification and thawing processes were performed with corresponding kits according to the manufacturer’s instructions. The blastocysts underwent artificial shrinkage before freezing. Embryos were thawed at an appropriate time in accordance with the individual transfer protocol, and then cultured until transfer. According to the previous studies (15, 18, 27) and the characteristics of the population in this study, cryopreservation duration was categorized as follows: 3–8 weeks (group 1, as the reference), 8–12 weeks (group 2), 12–26 weeks (group 3), 26–52 weeks (group 4), and >52 weeks (group 5).

Participants underwent a natural, programmed, or mild stimulation cycle regimen for endometrial preparation. The natural cycle is recommended for patients with normal ovulation and regular menstrual cycles. On the 10th day of menstruation, patients were monitored for ovulation using ultrasound. Dydrogesterone was administered for luteal phase support after ovulation. Programmed and mild stimulation cycle regimen are suitable for patients with irregular menstruation or a history of anovulation. For the programmed cycle regimen, oral oestradiol valerate was administered from day 1–3 of menstrual cycle at a dose of 4–8mg daily. When the endometrial thickness ≥7mm, dydrogesterone was added (28). For the mild stimulation cycle, gonadotropins, clomiphene citrate, or letrozole was started on day 2–3 of menstrual cycle. Frequent vaginal ultrasonography combined with serum endocrine assessment was used to monitor the follicles. When the diameter of the leading follicle >17 mm, hCG is administered. After the endometrium preparation, the frozen embryos were thawed and transferred. A serum β-hCG test was performed on day 14 after embryo transfer, and β-hCG level >30 mIU/mL was considered indicative of biochemical pregnancy. The presence of an intrauterine embryo sac confirmed *via* ultrasound 28 days after transfer was considered indicative of clinical pregnancy, while an ultrasound documented gestational sac outside the uterine cavity or pathologic evidence of an extrauterine pregnancy was considered as ectopic pregnancy. Live birth was defined as the delivery of a live-born infant. Miscarriage was defined as loss of pregnancy prior to 28 weeks of gestation.

### Statistical Analysis and Power Calculations

We performed an appropriate sample size estimation before determining the study subjects. In our previous multicenter retrospective study, the live birth rate was 38.8% (22). We estimated live birth rates in Group 2–Group 5 with reference to the study conducted by Li et al. (19). Assuming a live birth rate of 38.8% in Group 1, 35.9% in Group 2 and Group3, 34.2% in Group 4, and 28.8% in the Group5, power analysis showed that 17,661 subjects would be needed in total to achieve an 80% power to detect such a difference at a 95% confidence level. The demographic and clinical characteristics of the study cohort were presented as mean ± SD or frequency as percentage. Differences between groups were compared using Kruskal-Wallis tests for continuous variables and Chi-square tests or Fisher’s exact test for categorical variables. Reproductive outcomes, including biochemical pregnancy, clinical pregnancy, live birth, ectopic pregnancy, and miscarriage, were treated as binary variates. Poisson regression with robust variance estimation was fitted for biochemical pregnancy, clinical pregnancy, and live birth because of their high prevalence (29). Multivariable logistic regression analysis was performed to explore the associations of storage duration with ectopic pregnancy and miscarriage. The potential confounding factors were adjusted in models according to previous studies, including maternal age at oocyte pick-up (OPU) (continuous), body mass index (BMI) (continuous), reproductive center, infertility type (primary or secondary), cause of infertility (female factors, male factors, both, or unexplained), duration of infertility (continuous), endometrium preparation regimen (natural, programmed, or ovarian stimulation), oocyte yield (continuous), and the stage (cleavage or blastocyst), quality (high-quality or not), and number (continuous) of transferred embryos (19, 30, 31). Tests for overall linear trends were conducted using the median concentration in each group as a continuous variable.

Further stratification analyses were performed based on significant variables (*p <* 0.05) in multivariate models, including maternal age at OPU, the number of oocytes retrieved, and the stage, number, and quality of embryos transferred. All statistical analyses were performed using IBM SPSS Statistics 22.0 software (IBM Corp., Armonk, NY, USA). A two-tailed *p-*value < 0.05 was considered significant.

## Results

### Characteristics of the Study Population

**Figure 1:**
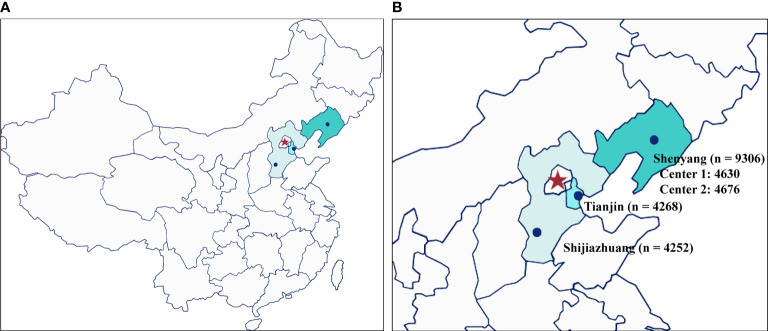
The geographical distribution and number of participants of the four different fertility centers. **(A)** overall location distribution; **(B)** the number of participants from the four reproductive centers included in this study.

A total of 17,826 women from four reproductive centers, who underwent their first FET cycle following the freeze-all strategy, were recruited in this large retrospective study. Of these, 9306 patients were from two reproductive center centers in Shenyang, 4268 were from Tianjin, and 4252 were from Shijiazhuang (**Figure 1**). Baseline and clinical information of the study population are summarized in **Table 1**. The mean age at OPU of the study population was 30.97 ± 4.59 years, the mean age at FET was 31.43 ± 4.64 years, and the mean BMI was 22.98 ± 3.43 kg/m^2^. The majority (99.2%) of these women were non-smokers. A total of 60.9% of women suffered from primary infertility. The mean duration of infertility was 4.37 ± 3.14 years, and nearly half of the couples sought infertility treatment due to female factors. Except for smoking status, there were significant differences in other characteristics across all groups. In terms of clinical information, the mean embryo cryopreservation time in this study was 13.53 ± 12.81 weeks and 92.8% of patients underwent their first FET within 26 weeks, with clinical pregnancy and live birth rates of 58.0% and 46.8%, respectively.

**Table 1:** Demographic and clinical characteristics of the study population.

	Total	Group 1	Group 2	Group 3	Group 4	Group 5	*p*-value
		3–8 weeks	8–12 weeks	12–26 weeks	26–52 weeks	>52 weeks	
Number	17,826	4734	6779	5028	958	327	
Cryopreserved time (weeks)	13.53 ± 12.81	5.68 ± 1.82	10.08 ± 1.13	16.84 ± 3.58	35.95 ± 7.24	82.12 ± 32.20	
Age at OPU (years)	30.97 ± 4.59	31.13 ± 4.63	30.50 ± 4.32	31.00 ± 4.62	32.58 ± 5.07	33.03 ± 5.42	<0.001**
Age at FET (years)	31.43 ± 4.64	31.37 ± 4.67	30.96 ± 4.35	31.52 ± 4.67	33.36 ± 5.11	34.82 ± 5.42	<0.001**
BMI (kg/m^2^)	22.98 ± 3.43	23.09 ± 3.44	22.79 ± 3.39	23.08 ± 3.51	23.19 ± 3.38	23.19 ± 3.53	<0.001**
Smoking status:							0.340
Smoker	148 (0.8%)	29 (0.6%)	61 (0.9%)	48 (1.0%)	7 (0.7%)	3 (0.9%)	
Non-smoker	17678 (99.2%)	4705 (99.4%)	6718 (99.1%)	4980 (99.0%)	951 (99.3%)	324 (99.1%)	
Infertility type:							<0.001**
Primary infertility	10860 (60.9%)	2856 (60.3%)	4197 (61.9%)	3103 (61.7%)	537 (56.1%)	167 (51.1%)	
Secondary infertility	6966 (39.1%)	1878 (39.7%)	2582 (38.1%)	1925 (38.3%)	421 (43.9%)	160 (48.9%)	
Duration of infertility (years)	4.37 ± 3.14	4.19 ± 3.06	4.31 ± 3.02	4.45 ± 3.19	4.88 ± 3.65	5.51 ± 3.75	<0.001**
Infertility cause:							<0.001**
Female factor	9466 (53.1%)	2767 (58.4%)	3403 (50.2%)	2601 (51.7%)	527 (55.0%)	168 (51.4%)	
Male factor	2200 (12.3%)	474 (10.0%)	944 (13.9%)	642 (12.8%)	99 (10.3%)	41 (12.5%)	
Both	4861 (27.3%)	988 (20.9%)	2006 (29.6%)	1482 (29.5%)	279 (29.1%)	106 (32.4%)	
Unexplained	1299 (7.3%)	505 (10.7%)	426 (6.3%)	303 (6.0%)	53 (5.6%)	12 (3.7%)	
Number of oocytes retrieved	17.64 ± 9.40	15.97 ± 9.08	19.18 ± 8.87	17.90 ± 9.73	15.00 ± 10.34	13.78 ± 9.70	<0.001**
Fertilization method:							<0.001**
IVF	10976 (61.6%)	2932 (61.9%)	4107 (60.6%)	3086 (61.4%)	629 (65.6%)	222 (67.9%)	
ICSI	5564 (31.2%)	1397 (29.5%)	2171 (32.0%)	1612 (32.0%)	287 (30.0%)	97 (29.7%)	
IVF + ICSI	1286 (7.2%)	405 (8.6%)	501 (7.4%)	330 (6.6%)	42 (4.4%)	8 (2.4%)	
Endometrium preparation regimen:							<0.001**
Natural cycle	4119 (23.1%)	796 (16.8%)	2017 (29.8%)	1055 (21.0%)	193 (20.1%)	58 (17.7%)	
Programmed cycle	13213 (74.1%)	3875 (81.9%)	4620 (68.1%)	3759 (74.8%)	706 (73.7%)	253 (77.4%)	
Minimal ovarian stimulation cycle	494 (2.8%)	63 (1.3%)	142 (2.1%)	241 (4.2%)	59 (6.2%)	16 (4.9%)	
Number of embryos transferred	1.67 ± 0.47	1.74 ± 0.44	1.61 ± 0.49	1.68 ± 0.47	1.72 ± 0.45	1.66 ± 0.47	<0.001**
Stage of transferred embryos:							<0.001**
Cleavage stage	12344 (69.2%)	3456 (73.0%)	4373 (64.5%)	3558 (70.8%)	718 (74.9%)	239 (73.1%)	
Blastocyst stage	5482 (30.8%)	1278 (27.0%)	2406 (35.5%)	1470 (29.2%)	240 (25.1%)	88 (26.9%)	
Biochemical pregnancy	11129 (62.4%)	2999 (63.4%)	4415 (65.1%)	3047 (60.6%)	499 (52.1%)	169 (51.7%)	<0.001**
Clinical pregnancy	10332 (58.0%)	2776 (58.6%)	4134 (61.0%)	2817 (56.0%)	454 (47.4%)	151 (46.2%)	<0.001**
Live birth	8350 (46.8%)	2245 (47.4%)	3395 (50.1%)	2247 (44.7%)	351 (36.6%)	112 (34.3%)	<0.001**
Ectopic pregnancy	175 (1.0%)	46 (1.0%)	64 (0.9%)	48 (1.0%)	13 (1.4%)	4 (1.2%)	0.710
Miscarriage	1672 (9.4%)	457 (9.7%)	629 (9.3%)	476 (9.5%)	80 (8.4%)	30 (9.2%)	0.781

Data were described as mean ± SD or N (%).

SD, standard deviation; OPU, oocyte pick-up; FET, frozen-thawed embryo transfer; BMI, body mass index; IVF, in vitro fertilization; ICSI, intracytoplasmic sperm injection.

**P < 0.01.

### Cryopreservation Duration Negatively Affects IVF Outcomes

The results of multivariable regression analysis indicated that cryopreservation duration was negatively associated with the likelihood of biochemical pregnancy, clinical pregnancy, and live birth (*p <* 0.001), while not with ectopic pregnancy and miscarriage (**Table 2**).

**Table 2:** Associations between cryopreservation time and pregnancy outcomes.

	Group 2	*p_2vs.1_*	Group 3	*p_3vs.1_*	Group 4	*p_4vs.1_*	Group 5	*p_5vs.1_*	*p*-trend
Biochemical pregnancy									
Unadjusted RR (95% CI)	1.028 (1.000,1.057)	0.051	0.957 (0.927,0.987)	0.005**	0.822 (0.771,0.877)	<0.001**	0.816 (0.733,0.908)	<0.001**	<0.001**
Adjusted RR (95% CI)	0.987 (0.959,1.016)	0.370	0.935 (0.906,0.965)	<0.001**	0.839 (0.788,0.894)	<0.001**	0.859 (0.775,0.952)	0.002**	<0.001**
Clinical pregnancy									
Unadjusted RR (95% CI)	1.040 (1.009,1.072)	0.012*	0.955 (0.923,0.989)	0.009**	0.808 (0.753,0.868)	<0.001**	0.787 (0.699,0.887)	<0.001**	<0.001**
Adjusted RR (95% CI)	0.994 (0.964,1.026)	0.709	0.933 (0.902,0.966)	<0.001**	0.829 (0.773,0.888)	<0.001**	0.836 (0.746,0.938)	0.001**	<0.001**
Live birth									
Unadjusted RR (95% CI)	1.056 (1.016,1.097)	0.005**	0.942 (0.903,0.984)	0.007**	0.773 (0.707,0.844)	<0.001**	0.722 (0.620,0.842)	<0.001**	<0.001**
Adjusted RR (95% CI)	0.996 (0.958,1.036)	0.855	0.916 (0.877,0.956)	<0.001**	0.795 (0.729,0.868)	<0.001**	0.776 (0.669,0.900)	<0.001**	<0.001**
Miscarriage									
Unadjusted OR (95% CI)	0.957 (0.843,1.086)	0.498	0.979 (0.855,1.120)	0.754	0.853 (0.665,1.093)	0.209	0.945 (0.642,1.393)	0.776	0.411
Adjusted OR (95% CI)	0.972 (0.852,1.108)	0.671	0.977 (0.851,1.122)	0.741	0.863 (0.672,1.1.09)	0.230	0.957 (0.647,1.414)	0.822	0.427
Ectopic pregnancy									
Unadjusted OR (95% CI)	0.971 (0.664,1.421)	0.881	0.982 (0.654,1.475)	0.931	1.402 (0.754,2.605)	0.285	1.262 (0.452,3.528)	0.657	0.486
Adjusted OR (95% CI)	1.067 (0.721,1.580)	0.744	0.991 (0.655,1.499)	0.967	1.394 (0.746,2.606)	0.348	1.309 (0.465,3.684)	0.649	0.522

The models were adjusted by maternal age at OPU, BMI, reproductive center, infertility type, infertility cause, infertility duration, endometrium preparation regimen, the number of oocytes retrieved, and the stage, number, and quality of embryos transferred. Group 1: 3–8 weeks; Group 2: 8–12 weeks; Group 3: 12–26 weeks; Group 4: 26–52 weeks; Group 5: > 52 weeks.

*P < 0.05, **P < 0.01.

### Stratification Analysis

We further conducted the stratification analysis according to the stage, number, and quality of embryos transferred. Since the number of cycles included in group 5 was too small, we excluded these cycles to ensure statistical performance across subgroups. The results of the stratification analysis indicated that the freezing duration did not affect the pregnancy outcomes in the cycles that transferred cleavage-stage embryos, regardless of the embryo quality (**Table 3**). However, frozen storage time of high-quality blastocysts was negatively correlated with FET outcomes in the group with blastocysts transferred (**Table 4**). Notably, in the cases of a high-quality plus a non-quality blastocyst transferred, storage time was not significantly correlated with pregnancy outcome.

**Table 3:** The results of stratification analyses assessing the associations between cryopreservation time and pregnancy outcomes based on the number and quality of cleavage embryos transferred.

	Biochemical pregnancy	Clinical pregnancy	Live birth
Single cleavage embryo transferred (n = 1231)		
High-quality (n = 1077)		
Group 1 (n = 211)	Reference	Reference	Reference
Group 2 (n = 471)	1.162 (0.954,1.415)	1.140 (0.920,1.413)	1.243 (0.952,1.622)
Group 3 (n = 323)	1.103 (0.895,1.360)	0.990 (0.785,1.250)	0.976 (0.731,1.304)
Group 4 (n = 72)	0.909 (0.630,1.311)	0.878 (0.587,1.312)	0.921 (0.564,1.503)
*p*-trend	0.890	0.321	0.283
No high-quality (n = 154)		
Group 1 (n =40)	Reference	Reference	Reference
Group 2 (n =46)	0.642 (0.306,1.350)	0.814 (0.338,1.959)	1.086 (0.374,3.155)
Group 3 (n =54)	0.447 (0.179,1.116)	0.529 (0.185,1.513)	0.799 (0.224,2.855)
Group 4 (n =14)	0.755 (0.273,2.090)	1.052 (0.358,3.096)	0.570 (0.104,3.138)
*p*-trend	0.213	0.500	0.415
Double cleavage embryo transferred (n = 10874)		
High-quality (n = 9214)		
Group 1 (n =2696)	Reference	Reference	Reference
Group 2 (n = 3313)	0.987 (0.949,1.026)	0.989 (0.947,1.032)	0.986 (0.935,1.039)
Group 3 (n = 2696)	0.962 (0.924,1.003)	0.961 (0.918,1.005)	0.950 (0.898,1.005)
Group 4 (n = 509)	0.932 (0.862,1.007)	0.922 (0.845,1.006)	0.904 (0.810,1.009)
*p*-trend	0.063	0.057	0.054
Single high-quality (n = 1245)		
Group 1 (n =377)	Reference	Reference	Reference
Group 2 (n = 401)	0.996 (0.875,1.133)	0.995 (0.866,1.142)	1.071 (0.901,1.273)
Group 3 (n = 377)	0.903 (0.789,1.034)	0.889 (0.768,1.028)	0.983 (0.822,1.174)
Group 4 (n = 90)	0.793 (0.615,1.023)	0.783 (0.597,1.026)	0.710 (0.492,1.024)
*p*-trend	0.074	0.061	0.116
No high-quality (n = 415)		
Group 1 (n =132)	Reference	Reference	Reference
Group 2 (n = 142)	1.045 (0.755,1.447)	1.097 (0.777,1.550)	1.201 (0.805,1.791)
Group 3 (n = 108)	1.039 (0.738,1.464)	0.993 (0.682,1.445)	1.051 (0.682,1.619)
Group 4 (n = 33)	0.782 (0.413,1.481)	0.641 (0.299,1.378)	0.287 (0.072,1.133)
*p*-trend	0.717	0.402	0.229

Data were presented as adjusted RR (95% CI).

The models were adjusted by maternal age at OPU, BMI, reproductive center, infertility type, infertility cause, infertility duration, endometrium preparation regimen, and the number of oocytes retrieved.

Group 1: 3–8 weeks; Group 2: 8–12 weeks; Group 3: 12–26 weeks; Group 4: 26–52 weeks; Group 5: > 52 weeks.

**Table 4:** The results of stratification analyses assessing the associations between cryopreservation time and pregnancy outcomes based on the number and quality of blastocysts transferred.

	Biochemical pregnancy	Clinical pregnancy	Live birth
Single blastocyst transferred (n = 4506)			
High-quality (n = 4166)			
Group 1 (n = 837)	Reference	Reference	Reference
Group 2 (n = 2019)	0.996 (0.947,1.049)	0.987 (0.933,1.045)	0.958 (0.889,1.031)
Group 3 (n = 1142)	0.928 (0.873,0.985)*	0.911 (0.852,0.973)**	0.856 (0.784,0.934)**
Group 4 (n = 168)	0.758 (0.656,0.876)**	0.732 (0.625,0.858)**	0.769 (0.640,0.923)**
*p*-trend	<0.001**	<0.001**	<0.001**
No high-quality (n = 340)			
Group 1 (n = 156)	Reference	Reference	Reference
Group 2 (n = 90)	0.780 (0.597,1.018)	0.781 (0.568,1.074)	0.677 (0.451,1.016)
Group 3 (n = 76)	0.908 (0.713,1.157)	0.934 (0.695,1.254)	0.918 (0.620,1.360)
Group 4 (n = 18)	0.573 (0.296,1.111)	0.718 (0.366,1.405)	0.603 (0.249,1.461)
*p*-trend	0.076	0.306	0.264
Double blastocyst transferred (n = 868)			
**High-quality (n = 614)**			
Group 1 (n = 210)	Reference	Reference	Reference
Group 2 (n = 202)	0.945 (0.854,1.044)	0.969 (0.868,1.082)	0.994 (0.856,1.154)
Group 3 (n = 172)	0.804 (0.711,0.909)**	0.841 (0.738,0.959)**	0.768 (0.640,0.922)**
Group 4 (n = 30)	0.857 (0.687,1.068)	0.870 (0.683,1.107)	0.677 (0.460,0.995)*
*p*-trend	0.001**	0.009**	0.001**
Single high-quality (n = 103)			
Group 1 (n = 24)	Reference	Reference	Reference
Group 2 (n = 25)	0.666 (0.448,0.991)*	0.627 (0.406,0.967)*	0.872 (0.480,1.586)
Group 3 (n = 42)	0.786 (0.537,1.150)	0.801 (0.535,1.198)	0.890 (0.480,1.650)
Group 4 (n = 12)	0.534 (0.271,1.052)	0.549 (0.280,1.076)	0.702 (0.300,1.644)
*p*-trend	0.128	0.216	0.505
No high-quality (n = 151)			
Group 1 (n = 51)	Reference	Reference	Reference
Group 2 (n = 50)	0.925 (0.726,1.178)	0.952 (0.717,1.264)	0.951 (0.628,1.442)
Group 3 (n = 38)	0.720 (0.511,1.014)	0.813 (0.568,1.163)	0.752 (0.441,1.281)
Group 4 (n = 12)	0.627 (0.308,1.279)	0.705 (0.341,1.458)	0.784 (0.326,1.888)
*p*-trend	0.053	0.172	0.304

Data were presented as adjusted RR (95% CI).

The models were adjusted by maternal age at OPU, BMI, reproductive center, infertility type, infertility cause, infertility duration, endometrium preparation regimen, and the number of oocytes retrieved.

Group 1: 3–8 weeks; Group 2: 8–12 weeks; Group 3: 12–26 weeks; Group 4: 26–52 weeks; Group 5: > 52 weeks.

*P < 0.05, **P < 0.01.

**Figure 2:**
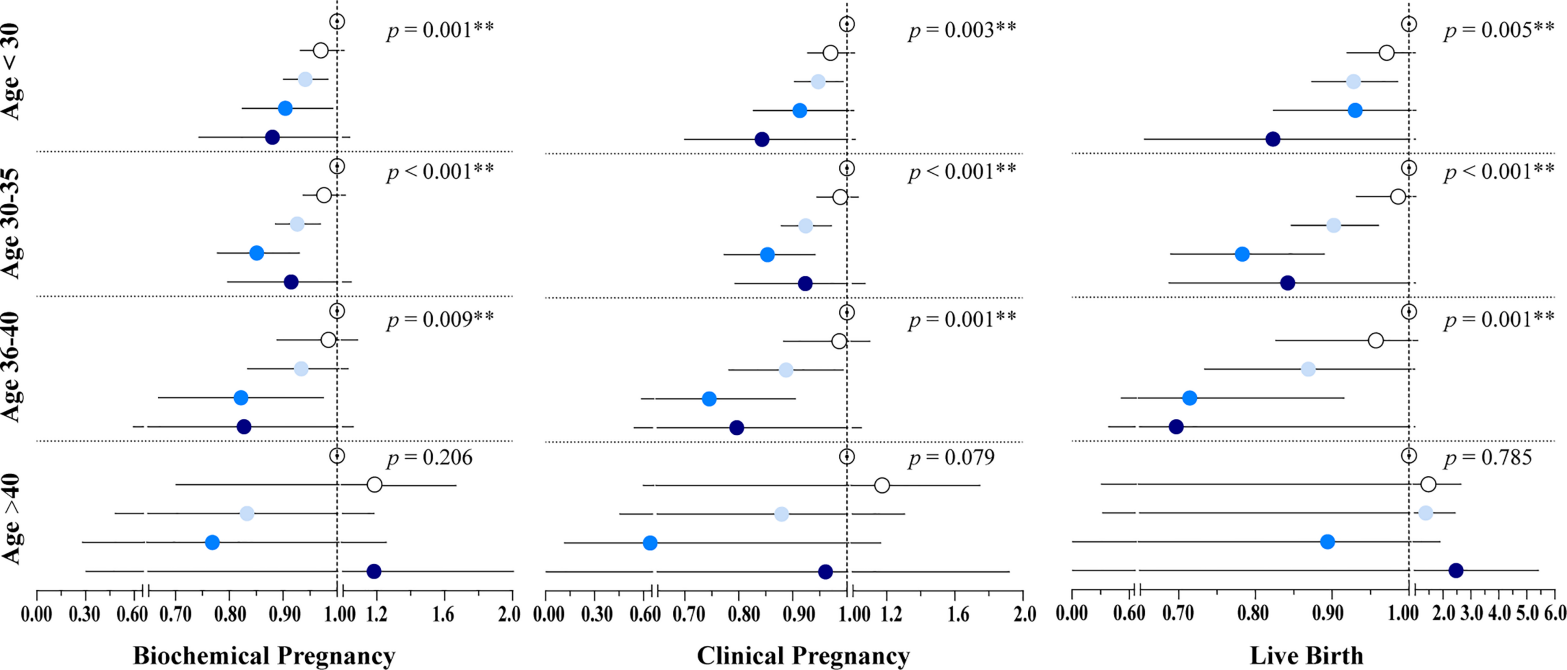
Results of the Poisson regression assessing the associations between cryopreservation time and pregnancy outcomes by age groups. The models were adjusted by maternal age at OPU, BMI, infertility type, infertility cause, infertility duration, endometrium preparation regimen, the number of oocytes retrieved, and the stage, number, and quality of embryos transferred. Hollow, white, light blue, blue, and dark blue circles represent the adjusted RR for Group1, Group 2, Group 3, Group 4, and Group 5, respectively. Line segments represent the 95% CI. Group 1: 3–8 weeks; Group 2: 8–12 weeks; Group 3: 12–26 weeks; Group 4: 26–52 weeks; Group 5: > 52 weeks. *P < 0.05, **P < 0.01.

In addition, we assessed the age-specific relationship between embryo cryopreservation duration and FET outcomes, stratifying participants into four groups according to age: <30 years, 30–35 years, 36–40 years, and >40 years. The results demonstrated that cryopreservation duration was negatively associated with pregnancy and live birth for women <40 years, with this trend being particularly significant among women aged 30–35 years (*p* < 0.001) (**Figure 2** and **Supplementary Table 1**), while no association was observed between cryopreservation duration and reproductive outcomes for women >40 years.

Finally, to explore the effect of cryopreservation duration on pregnancy outcomes in patients with different ovarian responsiveness, patients were classified into five groups according to the number of oocytes retrieved: low ovarian response (<4), suboptimal (4–9), normal (10–15), high (16–25), and ultra-high (>25) (32–34). The results of the multivariate model revealed a negative association of cryopreservation duration with pregnancy and live birth in women with a suboptimal or higher ovarian response (**Table 5**).

**Table 5:** The results of stratification analyses assessing the associations between cryopreservation time and pregnancy outcomes based on the number of oocytes retrieved.

	Biochemical pregnancy	Clinical pregnancy	Live birth
Oocytes retrieved < 4 (n = 978)			
Group 1 (n = 326)	Reference	Reference	Reference
Group 2 (n = 193)	0.958 (0.763,1.203)	0.921 (0.724,1.171)	0.978 (0.716,1.336)
Group 3 (n = 268)	0.932 (0.755,1.151)	0.825 (0.655,1.039)	0.784 (0.578,1.063)
Group 4 (n = 135)	0.789 (0.589,1.058)	0.765 (0.548,1.068)	0.738 (0.491,1.110)
Group 5 (n = 56)	0.827 (0.536,1.277)	0.719 (0.436,1.185)	0.670 (0.340,1.320)
*p*-trend	0.109	0.072	0.063
Oocytes retrieved 4-9 (n = 2789)			
Group 1 (n = 924)	Reference	Reference	Reference
Group 2 (n = 745)	1.011 (0.925,1.105)	1.006 (0.911,1.111)	0.990 (0.876,1.118)
Group 3 (n = 829)	0.986 (0.903,1.077)	0.985 (0.893,1.086)	0.958 (0.849,1.081)
Group 4 (n = 209)	0.822 (0.694,0.975)*	0.815 (0.675,0.983)*	0.713 (0.559,0.909)**
Group 5 (n = 82)	0.777 (0.595,1.013)	0.759 (0.551,1.046)	0.576 (0.375,0.884)*
*p*-trend	0.019*	0.012*	0.002**
Oocytes retrieved 10-15 (n = 3689)			
Group 1 (n = 1165)	Reference	Reference	Reference
Group 2 (n = 1346)	0.963 (0.908,1.021)	0.990 (0.928,1.056)	0.990 (0.913,1.073)
Group 3 (n = 942)	0.917 (0.859,0.980)*	0.920 (0.855,0.990)*	0.920 (0.840,1.008)
Group 4 (n = 178)	0.819 (0.712,0.942)**	0.813 (0.697,0.949)**	0.695 (0.564,0.856)**
Group 5 (n = 58)	0.764 (0.589,0.991)*	0.840 (0.647,1.091)	0.812 (0.588,1.121)
*p*-trend	<0.001**	0.001**	0.001**
Oocytes retrieved 16-25 (n = 7069)			
Group 1 (n = 1649)	Reference	Reference	Reference
Group 2 (n = 3039)	0.974 (0.934,1.016)	0.976 (0.931,1.022)	0.961 (0.906,1.019)
Group 3 (n = 2005)	0.939 (0.896,0.984)**	0.941 (0.894,0.991)*	0.919 (0.861,0.981)*
Group 4 (n = 293)	0.911 (0.830,1.001)	0.916 (0.827,1.015)	0.928 (0.818,1.053)
Group 5 (n = 83)	0.971 (0.831,1.136)	0.928 (0.774,1.111)	0.888 (0.702,1.123)
*p*-trend	0.005**	0.009**	0.011*
Oocytes retrieved > 25 (n = 3301)			
Group 1 (n = 670)	Reference	Reference	Reference
Group 2 (n = 1456)	0.973 (0.915,1.034)	0.993 (0.928,1.062)	1.030 (0.945,1.123)
Group 3 (n = 984)	0.908 (0.849,0.972)**	0.921 (0.855,0.991)*	0.912 (0.829,1.003)
Group 4 (n = 143)	0.862 (0.752,0.989)*	0.863 (0.742,1.003)	0.881 (0.732,1.061)
Group 5 (n = 48)	1.035 (0.873,1.226)	1.048 (0.870,1.263)	0.978 (0.753,1.271)
*p*-trend	0.006**	0.017*	0.016*

Data were presented as adjusted RR (95% CI).

The models were adjusted by maternal age at OPU, BMI, reproductive center, infertility type, infertility cause, infertility duration, endometrium preparation regimen, the number of oocytes retrieved, and the stage, number, and quality of embryos transferred.

Group 1: 3–8 weeks; Group 2: 8–12 weeks; Group 3: 12–26 weeks; Group 4: 26–52 weeks; Group 5: > 52 weeks.

*P < 0.05, **P < 0.01.

## Discussion

With the improvement of embryo freezing technology, the number of FET cycles performed worldwide has been increasing year by year. The elective freeze-all strategy has become a promising option for improving embryo transfer outcomes in specific populations (8), therefore, it is essential to evaluate the impact of cryopreservation duration on FET outcomes to guide clinical practice. In the present study, the embryo cryopreservation duration was negatively associated with the probability of biochemical pregnancy, clinical pregnancy, and live birth, especially in the high-quality blastocyst transferred subgroup. Moreover, for women <40 years old (especially 30-35 years old) and women with more than 3 oocytes retrieved, extended embryo frozen storage time significantly reduced the likelihood of pregnancy and live birth.

According to available studies, freezing may affect the embryonic cytoskeleton and DNA integrity, alter the miRNA transcriptome of embryos, and increase the incidence of imprinted gene mutations, subsequently contributing to impaired implantation potential of frozen-thawed embryos and inducing imprinting disorders (35–37). An *in vitro* study conducted by Mozdarani and Moradi (38) suggested that vitrification may decrease the viability of mouse embryos through chromosomal aberrations-mediated cell death, and the effects of which were dependent on the length of cryopreservation. Conversely, some animal studies have indicated that the cryopreservation duration of vitrified embryos did not significantly affect their survival rate, pregnancy rate, or live birth rate (39, 40).

In epidemiological studies, the effects of embryo cryopreservation duration on FET outcomes are controversial (19, 27). Aflatoonian et al. performed a retrospective study among women <39 years old, determining that cryopreservation duration did not affect FET outcomes (27). However, a single-center study by Li et al. with 24,698 patients demonstrated that prolonged frozen storage time reduced the likelihood of pregnancy and live birth (19), which is consistent with our findings. Notably, in this study, the elective freeze-all policy was used, on the contrary, Li et al.(19)’s reproductive center performed the freeze-all strategy in a non-elective manner. Therefore, the difference in characteristics of the study population might introduce bias. For instance, we noticed that the average number of oocytes retrieved in the population we included was higher than that in the population of the study by Li et al.(19) (17.64vs.10.81), which might be attributed to the different indications for the freeze-all strategy in the two studies. Moreover, The statistical method used in this study is different from that used by Li et al., in detail, modified Poisson regression was used to assess the impact of cryopreservation time on pregnancy and live birth in this study due to high prevalence, instead of the logistic regression used in the study by Li et al. (19). In summary, although the two studies have driven to similar conclusions, the results should be explained with caution, and future studies with prospective design should be conducted for further exploration.

In FET cycles, clinical pregnancy and live birth rates may vary depending on the developmental stage of the embryo transferred (41), hence it is necessary to analyze cleavage-stage embryos and blastocysts separately when exploring the effect of embryo cryopreserved time on FET outcomes. Li et al.’s study, which was based on an analysis of 786 vitrified-thawed cycles, reported no significant difference in FET outcomes following the transfer of cleavage-stage embryos cryopreserved within 5 years (15). Similarly, our study revealed that extended cryopreservation time had no significant impact on FET outcomes for cleavage-stage embryos.

Previous studies evaluating the effect of frozen storage time on the FET outcomes following blastocysts transferred have all reached similar conclusions (16, 18, 42). A small-scale study by Wirleitner et al. determined that prolonged cryopreservation of blastocysts did not affect pregnancy and live birth rates, while the study might be limited by its sample size and lack of adjustment for confounders (18). Another study by Sekhon et al. concerning the effect of blastocysts vitrified storage time on pregnancy outcomes obtained similar results (16). Notably, Sekhon et al.’s study included patients whose blastocysts underwent trophectoderm biopsy for aneuploidy screening before vitrification, however, whether this invasive procedure had any effect on the development and implantation potential of the blastocysts was not elucidated. Recently, Lee et al. revealed no significant association between cryostorage duration of blastocysts and the probability of clinical pregnancy and live birth (42). Nevertheless, an inverse association between extended storage time of high-quality blastocysts and the likelihood of clinical pregnancy and live birth was observed in the present study. One potential explanation for the discordant results is that Lee et al. (42) used slush nitrogen for embryo cryopreservation rather than traditional liquid nitrogen. The slush nitrogen could increase the cooling rate, lower the amount of cryoprotectant used, and reduce cryodamage to embryos, as reported. Moreover, although a significant correlation between the number and quality of blastocysts transferred and live birth was revealed in the study by Lee et al. (42), they did not perform relevant confounding adjustment or stratification analysis. In this study, we detected that the prolonged storage time did not affect FET outcomes following co-transfer of high-quality and non-high-quality blastocysts. In addition to possible bias introduced by small sample sizes in subgroups, this might be attributed to the interaction between embryos after co-transfer (43). Furthermore, we observed that vitrified storage time did not significantly correlate with FET outcomes for non-high-quality blastocysts, which was consistent with the findings of Ueno et al.’s study (17).

We also examined whether associations varied by maternal age at OPU and oocyte yield, and we detected that there was no significant correlation between frozen storage time and FET outcomes in women over 40 years old and women with less than 4 oocytes retrieved. Advanced maternal age is a key factor affecting the success of ART, as it can impair ovarian reserve, oocyte quality, and embryonic developmental potential. A recent study by Zhang et al. reported that several effector genes affecting quality were altered in oocytes from patients over 40 years old, including significant upregulation of oxidative stress-related genes (44). In women with advanced age, reactive oxygen species (ROS) clearance is impaired in oocytes, causing the accumulation of ROS in mitochondria, which in turn leads to mitochondrial dysfunction. Dysfunction of mitochondria and enhanced levels of oxidative stress in oocytes may cause DNA damage and chromosomal abnormalities, which potentially affect the developmental competency of oocytes, and even further impact the quality of the embryos formed from them (45, 46). In addition, we observed that the average age of women with fewer than 4 oocytes retrieved was significantly higher than that of women with suboptimal or higher ovarian response. Therefore, one potential explanation for the results derived from the stratification analyses is that the impact of cryopreservation on reproductive outcomes was attenuated by the impaired development potential secondary to the excessive oxidative stress level in the embryos themselves.

To our knowledge, this is the first large study to assess the effects of cryopreservation duration on FET outcomes across different geographic areas and populations, making our conclusions representative and generalizable. The longest duration of cryopreservation in this study was over four years, and our results mainly reflect the possible impact of short- to medium-term embryos cryopreservation on FET outcomes. Furthermore, maternal age at OPU, the stage, number, and quality of embryos transferred have been documented to be related to FET outcomes (31, 47). This study provides a comprehensive stratification analysis based on these factors for the first time, and hence further explores the impact of cryopreservation duration on reproductive outcomes in different subpopulations. In 2016, a large-scale randomized controlled trial concluded that frozen embryo transfer might benefit infertile women with PCOS, resulting in a higher live birth rate and lower risk of OHSS (10), which implies that the freeze-all strategy has a promising prospect. The conclusions of the present study, which were drawn from a multi-center, large-scale population, might guide clinicians on the timing of the first FET after the freeze-all strategy.

The present study also has several limitations. First, limited by the nature of its retrospective study design, although we set strict inclusion criteria, there were still unavoidable biases. Specifically, patients with a longer embryo cryostorage duration might undergo hysteroscopy or other medical treatments for embryo transfer. Therefore, these patients might be prone to have worse reproductive outcomes. However, these confounding factors could not be adjusted due to the limitation of data. Second, due to the lack of neonatal outcome data, this study did not include the comparison of perinatal outcomes and offspring follow-up.

## Conclusions

The results of this large, multicenter, retrospective study suggested that prolonged cryopreservation was associated with a lower probability of pregnancy and live birth. Stratification analyses indicated that the correlation was significant in the subpopulation with characteristics: (1) less than 40 years old, (2) more than 3 oocytes retrieved, and (3) only high-quality blastocysts transferred. Therefore, for patients who adopt a freeze-all strategy, early FET might be preferable to achieve an optimal outcome.

## Data Availability Statement

The original contributions presented in the study are included in the article/**Supplementary Material**. Further inquiries can be directed to the corresponding authors.

## Ethics Statement

The studies involving human participants were reviewed and approved by the Ethics Committee of Shengjing Hospital of China Medical University. Written informed consent for participation was not required for this study in accordance with the national legislation and the institutional requirements.

## Author Contributions

JT and YSZ: Conceptualization, Resources, Writing - Review and Editing, Supervision, Funding acquisition. GH, XW and HR: Resources, Supervision. XZ and SW: Conceptualization, Methodology, Formal analysis, Investigation, Writing - Original Draft. AY, YFZ, XB and LB: Data curation, writing. All authors contributed to the article and approved the submitted version.

## Funding

The study was granted from the National Key Research and Development Program (2018YFC1002105), the National Natural Science Foundation of China (82071601,61873257), the Key Research and Development Program of Liaoning Province (2018020222), the Central Government Special Fund for Local Science and Technology Development (2020JH6/10500006), the Major Special Construction Plan for Discipline Construction Project of China Medical University (3110118033), and the Shengjing Freelance Researcher Plan of Shengjing Hospital of China Medical University.

## Conflict of Interest

The authors declare that the research was conducted in the absence of any commercial or financial relationships that could be construed as a potential conflict of interest.

## Publisher’s Note

All claims expressed in this article are solely those of the authors and do not necessarily represent those of their affiliated organizations, or those of the publisher, the editors and the reviewers. Any product that may be evaluated in this article, or claim that may be made by its manufacturer, is not guaranteed or endorsed by the publisher.
