# Supplementary material for: Prolonged Cryopreservation Negatively Affects Embryo Transfer Outcomes Following the Elective Freeze-All Strategy: A Multicenter Retrospective Study
Source: Front Endocrinol (Lausanne). 2021 Sep 22;12:709648. doi: 10.3389/fendo.2021.709648 (PMC8493094; doi:10.3389/fendo.2021.709648)
Supplement: Supplementary file 1 [file Table_1.docx]

**Supplementary Table 1. Association between cryopreservation duration and pregnancy outcomes by age groups.**

|  | Biochemical pregnancy | Clinical pregnancy | Live birth |
| --- | --- | --- | --- |
| **Age < 30 (n = 6557)** | | | |
| Group 1 (n = 1786) | Reference | Reference | Reference |
| Group 2 (n = 2662) | 0.970 (0.931,1.010) | 0.970 (0.927,1.014) | 0.971 (0.919,1.026) |
| Group 3 (n = 1828) | 0.941 (0.900,0.984)** | 0.947 (0.902,0.994)* | 0.928 (0.873,0.986)* |
| Group 4 (n = 229) | 0.904 (0.823,0.993)* | 0.913 (0.826,1.009) | 0.930 (0.823,1.051) |
| Group 5 (n = 52) | 0.880 (0.743,1.042) | 0.843 (0.699,1.017) | 0.823 (0.655,1.034) |
| *p*-trend | 0.001** | 0.003** | 0.005** |
| **Age 30-35 (n = 8078)** | | | |
| Group 1 (n = 2109) | Reference | Reference | Reference |
| Group 2 (n = 3125) | 0.976 (0.936,1.017) | 0.988 (0.944,1.035) | 0.986 (0.931,1.045) |
| Group 3 (n = 2272) | 0.926 (0.885,0.970)** | 0.924 (0.878,0.972)** | 0.902 (0.846,0.961)** |
| Group 4 (n = 433) | 0.851 (0.777,0.931)** | 0.853 (0.772,0.942)** | 0.783 (0.689,0.890)** |
| Group 5 (n = 139) | 0.915 (0.796,1.051) | 0.923 (0.792,1.075) | 0.842 (0.687,1.032) |
| *p*-trend | ＜0.001** | ＜0.001** | ＜0.001** |
| **Age 36-40 (n = 2491)** | | | |
| Group 1 (n = 641) | Reference | Reference | Reference |
| Group 2 (n = 834) | 0.984 (0.888,1.089) | 0.986 (0.882,1.103) | 0.957 (0.826,1.110) |
| Group 3 (n = 720) | 0.930 (0.835,1.036) | 0.883 (0.783,0.996)* | 0.862 (0.737,1.008) |
| Group 4 (n = 211) | 0.812 (0.673,0.980)* | 0.734 (0.591,0.911)** | 0.695 (0.523,0.925)* |
| Group 5 (n = 85) | 0.805 (0.603,1.074) | 0.769 (0.555,1.065) | 0.649 (0.407,1.034) |
| *p*-trend | 0.009** | 0.001** | 0.001** |
| **Age > 40 (n = 700)** | | | |
| Group 1 (n = 198) | Reference | Reference | Reference |
| Group 2 (n = 158) | 1.120 (0.737,1.702) | 1.078 (0.649,1.790) | 1.168 (0.490,2.786) |
| Group 3 (n = 208) | 0.783 (0.508,1.208) | 0.810 (0.491,1.337) | 1.110 (0.480,2.563) |
| Group 4 (n = 85) | 0.664 (0.339,1.302) | 0.496 (0.201,1.224) | 0.511 (0.128,2.043) |
| Group 5 (n = 51) | 0.964 (0.431,2.156) | 0.640 (0.201,2.041) | 1.289 (0.285,5.830) |
| *p*-trend | 0.206 | 0.079 | 0.785 |

The models were adjusted by maternal age at OPU, BMI, reproductive center, infertility type, infertility cause, infertility duration, endometrium preparation regimen, the number of oocytes retrieved, and the stage, number, and quality of embryos transferred. Group 1: 3–8 weeks; Group 2: 8–12 weeks; Group 3: 12–26 weeks; Group 4: 26–52 weeks; Group 5: > 52 weeks.**P* < 0.05, ** *P* < 0.01.
