# Supplementary material for: Prolonged Cryopreservation Negatively Affects Embryo Transfer Outcomes Following the Elective Freeze-All Strategy: A Multicenter Retrospective Study
Source: Front Endocrinol (Lausanne). 2021 Sep 22;12:709648. doi: 10.3389/fendo.2021.709648 (PMC8493094; doi:10.3389/fendo.2021.709648)
Supplement: Supplementary file 2 [file Table_2.pdf]

Supplementary Table 1. Association between cryopreservation duration and pregnancy outcomes by age groups.

	Biochemical pregnancy	Clinical pregnancy	Live birth
**Age < 30 (n = 6557)**			
Group 1 (n = 1786)	Reference	Reference	Reference
Group 2 (n = 2662)	0.970 (0.931,1.010)	0.970 (0.927,1.014)	0.971 (0.919,1.026)
Group 3 (n = 1828)	0.941 (0.900,0.984)**	0.947 (0.902,0.994)*	0.928 (0.873,0.986)*
Group 4 (n = 229)	0.904 (0.823,0.993)*	0.913 (0.826,1.009)	0.930 (0.823,1.051)
Group 5 (n = 52)	0.880 (0.743,1.042)	0.843 (0.699,1.017)	0.823 (0.655,1.034)
p-trend	0.001**	0.003**	0.005**
**Age 30-35 (n = 8078)**			
Group 1 (n = 2109)	Reference	Reference	Reference
Group 2 (n = 3125)	0.976 (0.936,1.017)	0.988 (0.944,1.035)	0.986 (0.931,1.045)
Group 3 (n = 2272)	0.926 (0.885,0.970)**	0.924 (0.878,0.972)**	0.902 (0.846,0.961)**
Group 4 (n = 433)	0.851 (0.777,0.931)**	0.853 (0.772,0.942)**	0.783 (0.689,0.890)**
Group 5 (n = 139)	0.915 (0.796,1.051)	0.923 (0.792,1.075)	0.842 (0.687,1.032)
p-trend	＜0.001**	＜0.001**	＜0.001**
**Age 36-40 (n = 2491)**			
Group 1 (n = 641)	Reference	Reference	Reference
Group 2 (n = 834)	0.984 (0.888,1.089)	0.986 (0.882,1.103)	0.957 (0.826,1.110)
Group 3 (n = 720)	0.930 (0.835,1.036)	0.883 (0.783,0.996)*	0.862 (0.737,1.008)
Group 4 (n = 211)	0.812 (0.673,0.980)*	0.734 (0.591,0.911)**	0.695 (0.523,0.925)*
Group 5 (n = 85)	0.805 (0.603,1.074)	0.769 (0.555,1.065)	0.649 (0.407,1.034)
p-trend	0.009**	0.001**	0.001**
**Age > 40 (n = 700)**			
Group 1 (n = 198)	Reference	Reference	Reference
Group 2 (n = 158)	1.120 (0.737,1.702)	1.078 (0.649,1.790)	1.168 (0.490,2.786)
Group 3 (n = 208)	0.783 (0.508,1.208)	0.810 (0.491,1.337)	1.110 (0.480,2.563)
Group 4 (n = 85)	0.664 (0.339,1.302)	0.496 (0.201,1.224)	0.511 (0.128,2.043)
Group 5 (n = 51)	0.964 (0.431,2.156)	0.640 (0.201,2.041)	1.289 (0.285,5.830)
p-trend	0.206	0.079	0.785

The models were adjusted by maternal age at OPU, BMI, reproductive center, infertility type, infertility cause, infertility duration, endometrium preparation regimen, the number of oocytes retrieved, and the stage, number, and quality of embryos transferred. Group 1: 3–8 weeks; Group 2: 8–12 weeks; Group 3: 12–26 weeks; Group 4: 26–52 weeks; Group 5: > 52 weeks.

Supplementary Table 2. Association between cryopreservation time and pregnancy outcomes by insemination technique.

	Group 1	Group 2	Group 3	Group 4	Group 5	*p*-trend
**IVF (n = 10976)**						
Biochemical pregnancy						
Adjusted RR (95% CI)	Reference	0.991 (0.956,1.028)	0.934 (0.897,0.972)**	0.841 (0.778,0.910)**	0.890 (0.788,1.004)	<0.001**
Clinical pregnancy						
Adjusted RR (95% CI)	Reference	1.000 (0.961,1.040)	0.934 (0.894,0.976)**	0.843 (0.774,0.918)**	0.878 (0.768,1.004)	<0.001**
Live birth						
Adjusted RR (95% CI)	Reference	1.002 (0.952,1.054)	0.909 (0.860,0.962)**	0.810 (0.727,0.902)**	0.766 (0.638,0.921)**	<0.001**
**ICSI (n = 5564)**						
Biochemical pregnancy						
Adjusted RR (95% CI)	Reference	0.949 (0.902,1.000)	0.914 (0.865,0.966)**	0.854 (0.761,0.957)**	0.895 (0.802,0.999)*	<0.001**
Clinical pregnancy						
Adjusted RR (95% CI)	Reference	0.959 (0.906,1.014)	0.898 (0.845,0.955)**	0.776 (0.680,0.886)**	0.715 (0.563,0.907)**	<0.001**
Live birth						
Adjusted RR (95% CI)	Reference	0.949 (0.885,1.017)	0.885 (0.821,0.955)**	0.730 (0.620,0.859)**	0.764 (0.585,0.998)*	<0.001**

The models were adjusted by maternal age at OPU, BMI, reproductive center, infertility type, infertility cause, infertility duration, endometrium preparation regimen, the number of oocytes retrieved, and the stage, number, and quality of embryos transferred. Group 1: 3–8 weeks; Group 2: 8–12 weeks; Group 3: 12–26 weeks; Group 4: 26–52 weeks; Group 5: > 52 weeks
